# mycelyso – high-throughput analysis of *Streptomyces* mycelium live cell imaging data

**DOI:** 10.1186/s12859-019-3004-1

**Published:** 2019-09-04

**Authors:** Christian Carsten Sachs, Joachim Koepff, Wolfgang Wiechert, Alexander Grünberger, Katharina Nöh

**Affiliations:** 1 Institute of Bio- and Geosciences, IBG-1: Biotechnology, Forschungszentrum Jülich GmbH, Wilhelm-Johnen-Str., Jülich, 52425 Germany; 2 Multiscale Bioengineering, Bielefeld University, Universitätsstr. 25, Bielefeld, 33615 Germany; 3 Computational Systems Biotechnology (AVT.CSB), RWTH Aachen University, Aachen, 52074 Germany

**Keywords:** High-throughput analysis, Filamentous growth, Time-lapse imaging, Microfluidic cultivation, Streptomycetes, Mycelium network, Biotechnology, Hyphae tracking, Heterogeneity, Reproducible science, Open source

## Abstract

**Background:**

Streptomycetes are filamentous microorganisms of high biotechnological relevance, especially for the production of antibiotics. In submerged cultures, the productivity of these microorganisms is closely linked to their growth morphology. Microfluidic lab-on-a-chip cultivation systems, coupled with automated time-lapse imaging, generate spatio-temporal insights into the mycelium development of streptomycetes, therewith extending the biotechnological toolset by spatio-temporal screening under well-controlled and reproducible conditions. However, the analysis of the complex mycelial structure formation is limited by the extent of manual interventions required during processing of the acquired high-volume image data. These interventions typically lead to high evaluation times and, therewith, limit the analytic throughput and exploitation of microfluidic-based screenings.

**Results:**

We present the tool *mycelyso* (MYCElium anaLYsis SOftware), an image analysis system tailored to fully automated hyphae-level processing of image stacks generated by time-lapse microscopy. With *mycelyso*, the developing hyphal streptomycete network is automatically segmented and tracked over the cultivation period. Versatile key growth parameters such as mycelium network structure, its development over time, and tip growth rates are extracted. Results are presented in the web-based exploration tool *mycelyso Inspector*, allowing for user friendly quality control and downstream evaluation of the extracted information. In addition, 2D and 3D visualizations show temporal tracking for detailed inspection of morphological growth behaviors. For ease of getting started with *mycelyso*, bundled Windows packages as well as Docker images along with tutorial videos are available.

**Conclusion:**

*mycelyso* is a well-documented, platform-independent open source toolkit for the automated end-to-end analysis of *Streptomyces* image stacks. The batch-analysis mode facilitates the rapid and reproducible processing of large microfluidic screenings, and easy extraction of morphological parameters. The objective evaluation of image stacks is possible by reproducible evaluation workflows, useful to unravel correlations between morphological, molecular and process parameters at the hyphae- and mycelium-levels with statistical power.

**Electronic supplementary material:**

The online version of this article (10.1186/s12859-019-3004-1) contains supplementary material, which is available to authorized users.

## Background

Bacteria of the genus *Streptomyces* are important producers of a wide range of pharmaceuticals, such as antibiotics, anticancer agents, and immuno-suppressants [1, 2]. Owing to their secretion ability, *Streptomyces* are industrial production hosts for a large variety of enzymes and heterologous proteins [3]. Biotechnological processes with *Streptomyces* are predominantly performed in liquid (submerged) culture. Bioprocess optimization, although successful on a case-to-case basis, however, has hardly been found to be transferable between strains and target products [4]. The reason lies in the complex filamentous growth morphology (cf. Additional file 1: Figure S1) of these microbes which is intimately linked to production performances and cultivation stability. The life cycle of *Streptomyces* begins with spore germination, followed by growth of the initial germ tube, and outgrowth of hyphae which form a branched network, the mycelium (cf. Additional file 1: Figure S1). In submerged culture the mycelium tends to agglomerate, leading to the formation of “clumps” known as pellets. Pellets are orders of magnitude larger than those of the single hyphal elements: the length of hyphae is in the scale of micrometers while pellets are up to millimeters in diameter.

The study of *Streptomyces* and their intricate life cycle, growth morphology, and production performances remains a field of active research [2, 5]. Traditional microscopic observation parameters in *Streptomyces* cultivations are the mean overall growth kinetics of the developing mycelium as derived from the increase in pellet diameter. Hence, microscopy joint with image analysis techniques have been used since the 1970s to study filamentous microorganism growth behavior and kinetics [6]. However, for submerged cultures, these early approaches focused on snapshot analyses with pellet-level resolution.

Recently, microfluidic chip systems coupled with live-cell imaging unlocked the spatio-temporal screening of single microbial cell and colony development in monolayers under well-defined submerse and reproducible conditions. Thereby, this technology readily enables the in-depth study of morphological growth kinetics as well as the observation of the emerging heterogeneity of microbial cells at the single-cell level in hundreds to thousands picoliter-scaled growth chambers simultaneously [7]. In turn, this technology permits the massively parallel investigation of the developing mycelial network of *Streptomyces* in submerged culture with spatio-temporal resolution [8]. Precisely, the elongation kinetics of individual hypha become accessible along with the topology of the branching network allowing for the in-depth study of the mycelium fine structure development.

The high volumes of image data captured by time-lapse microscopy in microfluidic approaches, however, come with the burden of image analysis to extract meaningful bio(techno)logical metrics. To this end, specific software tools have been developed. Two recent approaches to pellet- or mycelium-level analysis of *Streptomyces* are the ImageJ [9] plugin *AnaMorf* [10] and a MATLAB & Mathematica solution by de Ulzurrun [11]. Although the latter can handle time-lapse data, neither this software nor *AnaMorf* has the capability to track mycelial development at the hyphae-level over time in an automated manner. More recently *MicrobeJ*, a general-purpose tool for microbial image analysis became available [12]. While the *MicrobeJ* software can track microscopic hyphal networks image by image, it is geared towards interactive image analysis, rather than high-throughput analysis as it is needed to comprehensively evaluate the high data volumes acquired by microfluidics-based screening platforms. Similarly, the MATLAB-based tool *DSeg*, aimed at time-lapse image data analysis of filamentous organisms including *Streptomyces*, involves manual steering of the analysis processes [13]. In the closely related field of fungi research, several tools have been developed for the analysis of snapshot data of mycelium structures. Here, two recent representative frameworks are *HyphaTracker* and *SParticle* [14, 15]. The ImageJ toolbox *HyphaTracker* for ascomycete analysis focuses on mycelium-level growth metrics while the ImageJ-macro package *SParticle* delivers growth measures with pellet- or mycelium-fragment-level resolution. These tools follow the same concepts as the streptomycete analysis tools and share similar characteristics.

Summarizing, employing existing tools for hyphae-level feature extraction and tracking from streptomycete time-lapse image sequences involves manual interactions which remain to be laborious and extremely time consuming, since automation aspects are insufficiently addressed by available image software packages. This lack of computational solutions hampers the exploitation of the unique possibilities offered by microfluidic single-cell technologies: the assessment of heterogeneity in a variety of growth measures and the ability to follow the development of exact mycelium structures over time, which later gives rise to pellet formation.

Here we describe the automated image processing suite *mycelyso* for analyzing mycelial network development of *Streptomyces*, with special focus on the extraction of growth metrics at the hyphae-level from time-lapse images. With *mycelyso*, growth metrics can be automatically extracted, in a reproducible manner, such as the hyphal growth unit (total mycelium length divided by tip count), hyphal elongation rates (change in hypha length over time), and whole mycelium elongation rates (change in total mycelium length over time). A viewer is developed, to provide an effective overview over the (both summary and detailed) results. Analysis results are stored in the HDF5 format to enable flexible and convenient post-processing and analysis, even in cases of large result sets. Diverse growth metrics can be extracted, with minimal manual burden and maximal reproducibility. The utility of *mycelyso* has been proven in a comprehensive screening study with *Streptomyces lividans* TK24 [16] where we analyzed the influence of different growth media compositions on the growth behavior from whole mycelium to individual hyphae-level.

## Implementation

The *mycelyso* analysis suite consists of two independent parts: the core command line tool analysis tool *mycelyso* for batch processing of phase contrast time-lapse image data, and the interactive web-based results viewer *mycelyso Inspector*. Both tools are written in the Python programming language, with a bundled package available for Windows, as well as a Docker image for quick and reproducible invocation. Documentation is available from the web page.

### The batch-processor *mycelyso*

*mycelyso* is a cross-platform command-line tool accepting plain TIFF, OME-TIFF [17], Zeiss CZI or Nikon ND2 image stacks. It uses the NumPy and SciPy libraries for computational efficiency, as well as the imaging processing library scikit-image [18, 19]. Data processing is pipelined, utilizing multiple CPU cores in parallel for processing speed. Various heterogeneous results (e.g., tabular data, graph data, segmented image data) are stored in the HDF5 format for easy and flexible post-analysis. *mycelyso* has been developed with fully automated batch processing in mind. Nevertheless, before starting a batch analysis process, adjustment of image analysis parameters might be indicated. To this end, *mycelyso* can be run in a preview mode, visualizing the binarization and the hyphae network in a per-frame manner. This way, the user can first visually assess and validate the analysis quality before launching batch processing of the image stacks.

#### Processing steps

##### Pre-processing.

**Fig. 1:**
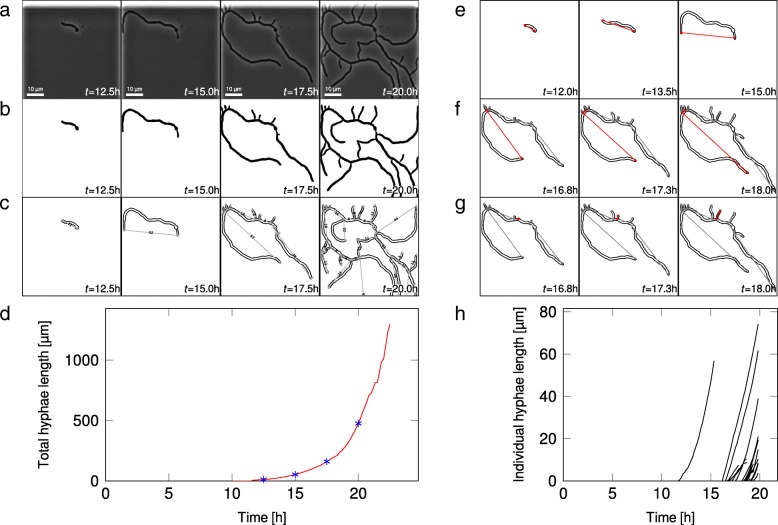
*Streptomyces lividans* TK24 cultured in planar growth chambers analyzed with *mycelyso*. **a** quadratic crops of the raw image sequence at four time points, showing mycelium outgrowth from the spore; **b** intermediate segmentation results of the raw images in **a**; **c** overlay of images and graph representation, with larger hyphae length annotated (in µm); the skeleton consisting of junction/tip points (circles) and connecting edges is shown in grey, numbers refer to skeleton (i.e. corresponding mycelium) lengths; **d** whole mycelium data as generated by *mycelyso*: total mycelium length over time as used for mycelium growth rate determination; **e-g** tracks of individual hyphae, denoted in red, with **e** showing elongation of the main hypha; **h** hyphae length increases over time allowing for assessment of heterogeneity in tip elongation rates

To prepare input data for *mycelyso*, it has to be registered (i.e. drift compensated). Depending on the on-chip growth structures present, it can be beneficial to crop images to a region of interest (ROI) to speed up processing and reduce possible artifacts. In microfluidic setups with rectangular growth chambers, such as described in Grünberger et al. [7], finding the region of interest with joint registration is automated in *mycelyso*, meaning that image stacks can be analyzed directly, as acquired from the microscope. Otherwise, cropping to a region of interest is to be performed beforehand, e.g. with standard tools like ImageJ [9]. The cropped image stack (Fig. 1a) is then background-corrected by subtraction of a blurred version of the image.

##### Binarization.

After the pre-processing, the image data is binarized. For this, *mycelyso* provides both, common local thresholding functions as well as a custom segmentation approach (an adaption of the binarization by Niblack [20]). Minor artifacts within the image, such as foreground or background speckles up to a certain size, are removed to alleviate proper downstream analyses, leading to cleaned binarized images (Fig. 1b).

##### Skeletonization.

The binary image is skeletonized using Zhang-Suen’s approach [21], i.e., it is transferred into a topology-preserving single pixel wide representation (Fig. 1c) of the hyphae.

##### Graph reconstruction.

The single pixel wide skeleton is then transformed into a graph representation (Fig. 1c) by identifying branching points, i.e. junctions and tips (end-points) and their connectivity [22]. Specifically, the graph representation is formed by junctions and tips of the mycelium structure (nodes) and their connections (edges) (Additional file 1: Figure S1). Edge weights of the graph are determined by the hypha length spanned. The connectivity of the graph is found by moving over the skeleton in a pixel-wise manner. The weights (lengths of the individual hyphal segments) are determined by adding the distances of the individual skeleton pixel coordinates, corrected by repetitive smoothing [23]. The processing steps to derive the graph reconstruction are performed for all images of the image stack, in a parallelized manner.

##### Tracking.

Tracking the growing mycelium at the hyphae level differs from other single-cell tracking problems, in that the hyphae have to be tracked individually within their mycelial context to produce hyphae-level metrics. Therefore, once the graphs of all images in a time-lapse image stack are generated, hyphal development is tracked with the nodes of the graph representations at hand. Here, junction points are tracked by nearest-neighbor matching as they keep their position under planar growth conditions, delivering node correspondences between time frames (Fig. 1e-g). Tip tracking follows the same principle, but with a larger search radius, since the hyphae tips of *Streptomyces* grow by extension, thus moving forward during mycelial growth. To robustify the results, various sanity checks are applied while tracking the paths of individual hyphae. For instance, it is asserted that the paths remain connected by assuring they are connected components in the graphs in consecutive time points. Adjacent hyphae that cannot be separated in the binarized image (e.g., due to overgrowth), appear as a single edge in the graph, which might introduce “shortcuts” in the hypha tracks. To not generate artifactual growth metrics, affected hyphae are not tracked further. Once the tracking has been performed per growth chamber of the microfluidic chip (position), this information feeds in associated growth metrics, such as growth rate per position.

##### Growth metrics & result storage.

*mycelyso* provides plenty of growth and tracking metrics on hyphae, hyphae track, and position level (cf. Table 1), along various fits of time-resolved data to quickly extract growth kinetics. Values for all metrics as well as all intermediate results of the processing pipeline, such as binarization and skeletonization results, GraphML graph representations, results per position and hyphae (Fig. 1d,h) are stored in an HDF5 file, along with the processing parameters.

**Table 1:** Metrics automatically collected and derived from the image data, as stored tabularly in the HDF5 file

Level	Metric	Fits
*Per position and time*	Image dimensions	
	Crop values	
	Covered area	Linear/logarithmic^1^, raw/optimized^2^
	Total mycelium length	Linear/logarithmic^1^, raw/optimized^2^
	Branching point count	Linear/logarithmic^1^, raw/optimized^2^
	Tip count	Linear/logarithmic^1^, raw/optimized^2^
*Per hypha track*	Start/mid/end time point	
	Duration spanned	
	Count of time points spanned	
	Minimum/maximum length	
	Minimum/maximum node hop count	
*Per hypha and time*	Time point	
	Length	Absolute/relative^3^, Linear/logarithmic^1^, Raw/optimized^2^
	Node identifiers	
	Distance in node hops	
	Node identifiers of the successor segment	

^1^Logarithmic fit: data are transformed by taking the natural logarithm and fitted with linear regression

^2^Optimized fit: data are fitted within the widest continuous support with *R*^2^>0.9

^3^Relative length: length measurement is taken relative to the minimum hypha length of the track

##### Visualization & post-analysis.

For visualization and exploration of the data contained in the HDF5 file, the viewer *mycelyso Inspector* has been developed. With *mycelyso Inspector*, growth rates on hyphae- and mycelium-level can be viewed (cf. next paragraph). Alternatively, two Jupyter notebooks are included in the source code repository, which show additional ways to analyze the raw data stored in the HDF5 file. One notebook shows how to explore individual hyphae lengths and graph representations. The second notebook exemplifies how length-based growth rates can be determined using custom growth models by utilizing a third-party fitting tool.

### The viewer *mycelyso Inspector*

**Fig. 2:**
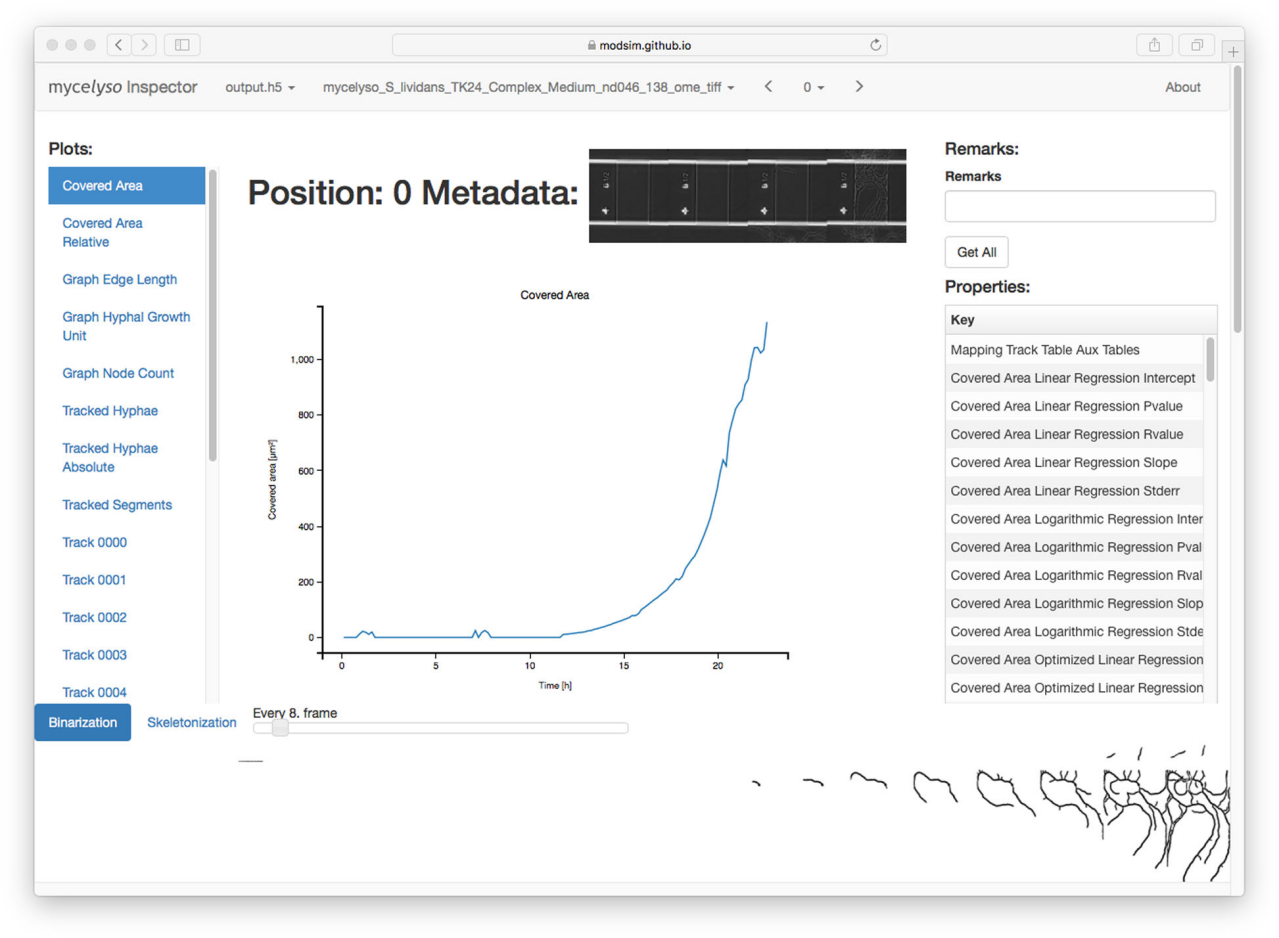
*mycelyso Inspector*, web-based inspection tool for raw data exploration and spatio-temporal result visualization, presenting various mycelium-level information (total hyphae length), hyphae-level individual track information, and reconstructed 2D/3D tracking graphs (cf. Fig. 1, Additional file 2: Figure S2)

Automated single-cell experiments generate high-volume data sets. To overview the collected data, a tailored web-based interactive data exploration tool, *mycelyso Inspector*, has been developed (Fig. 2). *mycelyso Inspector* enables visual quality control to detect poor quality results (due to technical or biological artifacts) which is essential before reliable interpretation and downstream analysis. To make high-volume data sets easily accessible, the web-based interactive data exploration tool *mycelyso Inspector* relies on a HTML5-based front end being served by a Python-based back end. The different kinds of data stored in the HDF5 files can be viewed either in a tabular or graphical (i.e., plots) manner, and individual results can directly be saved in tab-separated values (TSV) format. Graph data structures and intermediate segmentation results are visualized for checking the processed results. In addition, 3D views visualizing the growing hyphal network are presented for a quick overview of the spatio-temporal hyphal development and the analysis quality (Additional file 2: Figure S2).

## Results

*mycelyso* has been used to analyze a large-scale screening study to quantify the impact of six different growth media compositions on the hyphae-level growth behavior of *Streptomyces lividans* TK24 [16]. For the description of the imaging conditions and the biotechnological findings derived from the data, the reader is referred to the study [16]. Within this software publication, we focus on presenting performance measures for the *mycelyso* software. A representative image stack acquired in the study, growth in complex medium, is available as example data set at DOI: https://dx.doi.org/10.5281/zenodo.376281. The stack contains 136 time points, and was used to assess the performance of the software. The processing of the file (1.4 GiB) took 52.5 ± 1.2 s on a quad core Intel Core i7 4790 (3.6 GHz) with 32 GB RAM under Linux (approx. 2.5 frames per second, parallelized). Automated analysis directly yields important second-order metrics, such as the total mycelium growth rate, *μ*=0.45 h^−1^, derived from the mycelium length in one cultivation chamber (Fig. 1d), or the germination delay of 11.8 h, derived from the first occurring hypha among the tracked hyphae (Fig. 1h). These individual hyphae-level tracks further provide intra-mycelium growth parameters, such as a tip growth rates of 16.33±6.91 µ m·h^−1^. In comparison, the overall growth rate taking multiple on-chip positions (n=10) into account was determined to be 0.38±0.11 h^−1^ [16], and the germination delay 10.0±2.4 h.

## Discussion

Hyphae-level metrics unravel the precise kinetics of growth and response of the organism to the selected cultivation conditions, and provide insights into the formation of macroscopic phenotypes. In the larger study [16], from which the example dataset was taken, six different media compositions (one complex medium and five different minimal media) were imaged over time frames of 24–48 h. This setup yielded 675 image stacks in total. The image stacks were processed with *mycelyso* as described before and generated the biotechnological key parameters needed for the study in an automated manner. On this basis, we were able to draw statistically sound comparisons between the employed conditions without manual adaption of the processing. *mycelyso* contains specific developments for the task of streptomycete analysis, such as an adapted segmentation routine, as well as an individual hyphae-tracking approach for taking the mycelial network (graph) structure into account.

## Conclusions

*mycelyso* is an open source, ready-to-use platform independent analysis software for all steps of the analysis of time-lapse image stacks of growing hyphae networks of microfluidic *Streptomyces* cultivations. The end-to-end suite is designed for high-throughput hyphae tracking in a fully automated manner, and comes with the dedicated viewer *mycelyso Inspector* for visualizing the extracted results. *mycelyso* was successfully applied to evaluate a microfluidic *Streptomyces* growth study ([16], a screening of growth behavior under different media compositions, compared to micro-titer plate cultivation), which encompassed automated processing of ≈680 GiB image data, more than 600 individual image stacks consisting of 100-200 time points each. Microfluidic systems in combination with time-lapse microscopy and *mycelyso*, thus, facilitate the reproducible routine study of various aspects in growth and development of *Streptomyces* [2] and pave the way for further screening applications.

Data at hyphae-level is valuable to foster the understanding of growth mechanisms of *Streptomyces* and their relation to production performances, e.g., for validating morphology models that aim to predict the effect of changes in media compositions on bioreactor-scale cultivations. Although designed for the analysis of *Streptomyces*, the well-documented and modular software may be a useful starting point for analyses of other filamentous growing organisms.

## Availability and requirements

**Project name:** mycelyso

**Project home page:** https://github.com/modsim/mycelyso

**Operating systems:** Platform independent, Windows bundles and Docker images available additionally.

**Programming language:** Python 3

**Other requirements:** NumPy, SciPy, scikit-image, matplotlib, pandas, networkx, and other various dependency libraries, which can automatically be installed using setup.py.

**License:** BSD

**Any restrictions to use by non-academics:** none

## Additional files

Additional file 1 Figure S1. Schematic representation of “microscopic” multi-cellular mycelium morphology. (PDF 41 kb)

Additional file 2 Figure S2. *mycelyso Inspector*: 2D/3D in-browser visualization. (PDF 464 kb)

## Data Availability

A demo dataset is available at DOI 10.5281/zenodo.376281.
