# Supplementary material for: mycelyso – high-throughput analysis of Streptomyces mycelium live cell imaging data
Source: BMC Bioinformatics. 2019 Sep 4;20:452. doi: 10.1186/s12859-019-3004-1 (PMC6727546; doi:10.1186/s12859-019-3004-1)
Supplement: Supplementary file 1 — Figure S1. Schematic representation of “microscopic” multi-cellular mycelium morphology. (PDF 41 kb) [file 12859_2019_3004_MOESM1_ESM.pdf]

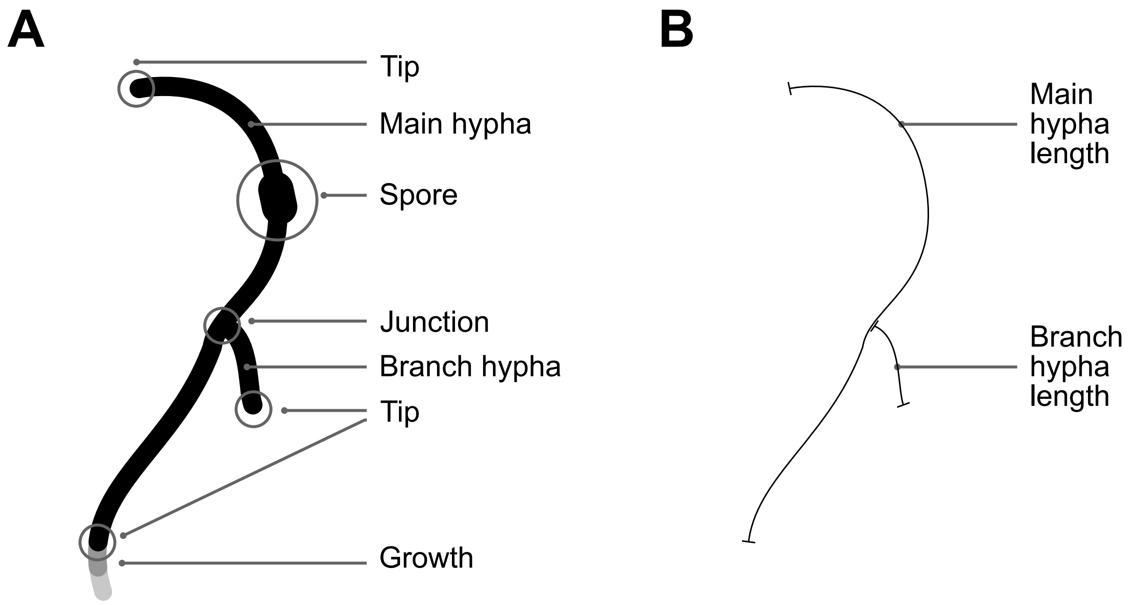

Figure S1 Schematic representation of “microscopic” multicellular mycelium morphology. A: Hyphal representation. Hyphal elements arise from the outgrowth of a single spore. After spore germination, the spore polarizes and forms a hypha which grows only at the tip (main hypha). During tip growth, new hyphae are formed (branch hypha), developing into simple branched networks which later form complex composite structures; **B**: Hyphal length representation.
