# Supplementary material for: mycelyso – high-throughput analysis of Streptomyces mycelium live cell imaging data
Source: BMC Bioinformatics. 2019 Sep 4;20:452. doi: 10.1186/s12859-019-3004-1 (PMC6727546; doi:10.1186/s12859-019-3004-1)
Supplement: Supplementary file 2 — Figure S2. mycelyso Inspector: 2D/3D in-browser visualization. (PDF 464 kb) [file 12859_2019_3004_MOESM2_ESM.pdf]

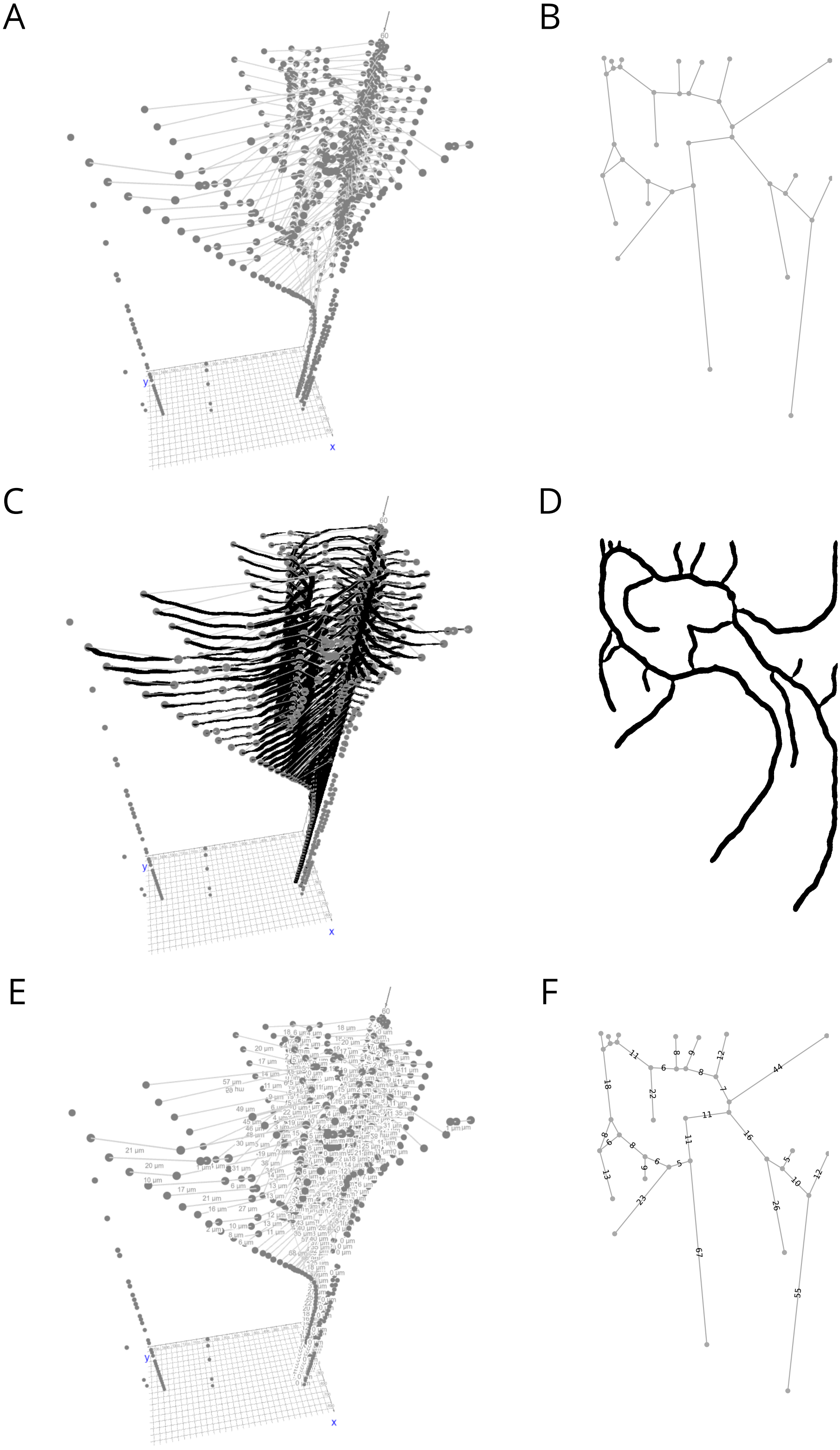

Figure S2 ***mycelyso Inspector*: 3D In-Browser Visualization.** A, C, E: The time-resolved, freely rotatable graph can be inspected in 3D. x-y plane: node positions of junctions and endpoints, z-axis: time step of image stack. B, D, F: Data of last frame (top-most in A, C, E) visualized in 2D. With A, B: Track graph; C, D: Track graph with overlaid segmentations; and, E, F: Track graph with hyphae length annotations.
